# Circulating T Cell Activation and Exhaustion Markers Are Associated With Radiation Pneumonitis and Poor Survival in Non-Small-Cell Lung Cancer

**DOI:** 10.3389/fimmu.2022.875152

**Published:** 2022-07-14

**Authors:** Janna Berg, Ann Rita Halvorsen, May-Bente Bengtson, Morten Lindberg, Bente Halvorsen, Pål Aukrust, Åslaug Helland, Thor Ueland

**Affiliations:** ^1^ Department of Medicine, Vestfold Hospital Trust, Tønsberg, Norway; ^2^ Department of Cancer Genetics, Institute for Cancer Research, Norwegian Radium Hospital, Oslo University Hospital, Oslo, Norway; ^3^ Department of Clinical Medicine, University of Oslo, Oslo, Norway; ^4^ Department of Oncology, Oslo University Hospital, Oslo, Norway; ^5^ Department of Medical Biochemistry, Vestfold Hospital Trust, Tønsberg, Norway; ^6^ Research Institute of Internal Medicine, Oslo University Hospital Rikshospitalet, Oslo, Norway; ^7^ Institute of Clinical Medicine, University of Oslo, Oslo, Norway; ^8^ Section of Clinical Immunology and Infectious Diseases, Oslo University Hospital Rikshospitalet, Oslo, Norway; ^9^ K.G. Jebsen Thrombosis Research and Expertise Center, University of Tromsø, Tromsø, Norway

**Keywords:** lung cancer, radiotherapy, stereotactic body radiation therapy, radiation pneumonitis, radiation-induced lung injury (RILI), blood biomarkers, t cell, leukocyte subsets

## Abstract

**Introduction:**

Persistent inflammation and immune activation in the lungs are associated with adverse outcomes such as radiation pneumonitis (RP) and poor survival in non-small-cell lung cancer (NSCLC) patients. However, it is unknown how this is reflected by leukocyte activation markers in serum.

**Objective:**

The aim was to evaluate the serum levels of activation of different leukocyte subsets and to examine those in relation to the pathogenesis of RP and survival in NSCLC.

**Methods:**

We analyzed the serum levels of MPO, sCD25, sTIM-3, sPD-L1, sCD14, sCD163, CCL19 and CCL21 in 66 inoperable NSCLC patients with stage IA-IIIA disease. The patients were treated with stereotactic body radiation therapy (SBRT) or concurrent chemoradiation therapy (CCRT), followed by regular blood sampling for 12 months after treatment and for 5 years for survival.

**Results:**

Nineteen (29%) patients developed RP, which occurred more frequently and earlier in patients receiving CCRT than in those receiving SBRT. Increases in sCD25, sTIM-3 and CCL21 levels were observed at the last 6 months of follow-up in patients who had RP after SBRT. Patients who had RP after CCRT had higher sTIM-3 levels during the first 3 months of follow-up. Baseline sCD25 was independently associated with both 2- and 5-year mortality outcomes, while baseline sTIM-3 was independently associated with 2-year mortality.

**Conclusion:**

We showed that T cell activation and exhaustion markers such as sCD25 and sTIM-3 are enhanced in patients developing RP and are associated with poor survival in NSCLC.

## Introduction

In 2020, lung cancer was the second most diagnosed cancer in the world and was the leading cause of cancer death. It is estimated that 2.2 million new cases occurred in the world in 2020 (1).

Radiation therapy is the most commonly used treatment modality for non-small-cell lung cancer (NSCLC) patients. It is administered for curative purposes as a solo treatment or in combination with surgery and/or chemotherapy for patients with early-stage or locally advanced stage (stage I-III) NSCLC. It can also be used as palliative treatment to prolong life and improve quality of life for patients with distant metastases (stage IV). Curative radiotherapy options are stereotactic body radiation therapy (SBRT) and concurrent chemoradiation therapy (CCRT). During the recent years, however, a number of novel treatment modalities has been developed such as check-point inhibitors and several tyrosine kinase inhibitors targeting specific genetic alterations.

The lung is a complex organ consisting of at least 40 different types of cells with distinct functions. This complexity is associated with impaired regeneration potential, and consequently, the lung is the organ most exposed to damage following various forms of radiation therapy (2–4). The risk of developing radiation-induced lung injury limits effective high-dose thoracic radiation therapy for early-stage and locally advanced NSCLC patients (5) (5). The reported incidence of radiation pneumonitis (RP) after SBRT for NSCLC varies from 2% to 47% (6–11), and the incidence after CCRT varies from 5% to 40% (12–17).

The tumour microenvironment (TME) plays an important role in tumour growth (18) and in the outcome of treatment, including affecting resistance to cancer treatment (19). TME of a solid tumour consists of tumour cells, local cells, infiltrating nontumour cells, molecules present in the vicinity of these cells and cells comprising the blood and lymph vessels. Radiotherapy affects cancer cells and the TME, in particular the tumour blood vessels and cells of the immune system.

Radiation induces reactive oxygen species and reactive nitrogen species (ROS and NGS), which cause damage to mitochondrial DNA and to the alveolar–capillary barrier, both of which are sensitive to the effects of ionizing radiation (20–22). One of the many effects of radiation is increased vascular permeability and exudation of proteins into the alveolar space, causing the apoptosis of alveolar type-I pneumocytes. This triggers an influx of inflammatory cells (e.g., neutrophils, macrophages, and lymphocyte subsets) from the peripheral and pulmonary vasculature that infiltrate the damaged lung. These cells are further activated by ROS and NGS as well as danger-associated molecular patterns (DAMPs), leading to the release of various inflammatory molecules (23–26) and contributing to altered tissue remodeling, fibrogenesis and local and systemic inflammation associated with the development of complications to radiotherapy (27, 28) and poor survival (29, 30). The radiation-induced release of cytokines and related molecules the first 24 hours after radiation might be an important contributor to RP (31–34).

While clinical outcome like survival is the most important parameter when evaluating novel treatment options, biomarkers are of importance in order to predict treatment responses and risk categories and even more importantly, to select correct treatment options and to discover pathways that are not modulated by the current treatment modalities.

The inflammatory response in the lungs causing the development of RP and fibrosis is still not well understood. The regulation and importance of the different inflammatory and immune-related mediators in the TME are at present not clear. In the present study, we examined the serum parameters of the activation of different leukocyte subsets, including myeloperoxidase (MPO) as a marker of neutrophil activation; soluble CD25 (sCD25), soluble T cell immunoglobulin mucin domain-3 (sTIM-3) and soluble programmed cell death 1 (sPD-1) as markers of T cell activation and exhaustion; and sCD14 and sCD163 as markers of monocyte/macrophage activation. We also analyzed the levels of the homeostatic chemokines CCL21 and CCL19 as mediators of lymphocyte trafficking. These markers were evaluated in relation to RP and survival after curative radiotherapy for early-stage and locally advanced NSCLC.

## Materials and Methods

### Trial Design

This is a prospective, longitudinal, clinical, single-institution (Vestfold Hospital Trust, Tønsberg, Norway) study for patients with early-stage and locally advanced stage (stage IA-IIIA) NSCLC (ClinicalTrials.gov NCT02428049).

### Patients

Eligible patients were > 18 years old and had early-stage or locally advanced-stage (stage IA-IIIA) NSCLC. Tumours were staged in accordance with the Union for International Cancer Control, Tumor, Node, Metastasis staging system 8th edition (TNM 8). Patients were examined with CT scans of the chest and abdomen and PET-CT, and all patients in stage IIIA were examined with brain MRI. Patients were technically resectable but deemed medically inoperable by a multidisciplinary tumour board, and the assignment was independent of the study. Patients were recruited from Vestfold Hospital Trust, Tønsberg, Norway, received SBRT or concomitant chemoradiotherapy at Oslo University Hospital, Radiumhospitalet, and underwent clinical follow-up at Vestfold Hospital Trust. A total of 66 patients were included in the study. Changes in pulmonary function, symptoms, and radiological signs of RP after SBRT have previously been studied in 44 of these patients (10).

### Ethics

All patients provided written informed consent. The study was conducted following legal and regulatory requirements as well as with the general principles outlined in the International Ethical Guidelines for Biomedical Research Involving Human Subjects (Council for International Organizations of Medical Sciences 2002) and the Declaration of Helsinki (World Medical Association 1996 and 2008). Regional Ethical Committee, REK nr. 2013/169/REK sør-øst D. The trial is registered with ClinicalTrials.gov (NCT02428049).

### Blood Sample Processing

Peripheral venous blood was collected with 4-mL Vacutainer tubes (BD Biosciences, San Diego, CA), kept in room temperature for coagulation for one hour and then spun at 1610 g for 10 minutes. Serum samples were stored immediately at –80°C in several aliquots in cryovials until analysis. Blood samples were collected before radiotherapy (baseline), on the last day of radiotherapy, at 1-1.5 months after treatment, and every 3 months thereafter until 12 months after radiotherapy. The samples were thawed only once.

### Enzyme Immuno-Assays

Serum levels of sCD14, sCD163, sCD25, sTIM-3, MPO, sPD-1, CCL19 and CCL21 (**Table 1**) were measured in duplicate by EIA using commercially available antibodies (R&D Systems, Minneapolis, MN) in a 384-format using a combination of a SELMA pipetting robot (Analytik Jena AG, Jena, Germany) and a BioTek dispenser/washer (BioTek Instruments, Winooski, VT). Absorption was read at 450 nm by using an EIA plate reader (BioTek Instruments) with wavelength correction set to 540 nm. Samples from a patient were run on the same 384-well plate, with controls randomly distributed on all plates; the intra- and interassay coefficients of variation were <10%.

**Table 1:** Markers included in the study.

Markers for	Protein short name	Protein full name
**T cells activation and exhaustion**	sTIM3	T cell immunoglobulin and mucin domain-containing protein 3
	sPD-1	Programmed cell death 1
	sCD25	Soluble interleukin-2 receptor alpha chain (IL-2Rα)
**Chemokine (chemotactic cytokines motif) family**	CCL19	Chemokine (C-C motif) ligand 19
	CCL21	Chemokine (C–C motif) ligand 21
**Neutrophil activation**	MPO	Myeloperoxidase
**Macrophage/monocyte activation**	sCD163	Cluster of differentiation 163
	sCD14	Cluster of differentiation 14

### Radiotherapy

Of the 66 patients in this study, 44 were treated with SBRT, and 22 were treated with CCRT. SBRT was administered as a total dose of 45–56 Gy in 3–8 fractions. The tumour was given an inhomogeneous dose where the prescribed dose encompassed the periphery of the planning target volume (PTV) and the maximum dose in the tumour was approximately 150% of the prescribed dose. Treatment planning was performed on an ordinary CT series. Respiratory-dependent tumour movement was visualized radiologically, and if more than 10 mm, abdominal compression was used to reduce it. This was applied for nine patients.

CCRT was administered with a radiation dose of 60-66 Gy and two courses of cisplatin and etoposide for 18 patients. Four patients were administered conventionally fractionated radiotherapy of 60-66 Gy alone.

### Follow-Up Specifications

Follow-up included a physical examination by a pulmonologist and pulmonary function evaluation at baseline, at 1-1.5 months after treatment, and every 3 months thereafter until 12 months after radiotherapy. CT scans were performed at all follow-up visits except at 1-1.5 months and 9 months when chest X-rays were carried out. Patients with symptoms were also referred for CT scans at 1-1.5 months and 9 months. After the first year, CT scans of the chest, a physical examination by a pulmonologist, spirometry, and determination of the DLCO according to national guidelines were performed two times the second year and yearly for the next three years.

### Grading of RP

The patients’ symptoms were graded according to the Common Terminology Criteria for Adverse Events (CTCAE). Radiological changes were graded according to the European Organization for Research and Treatment of Cancer and Late Effects Normal Tissues-Subjective, Objective, Management, Analytic (EORTC/LENT-SOMA). Based on the CTCAE and EORTC/LENT-SOMA grading, the patients were divided into the following 2 groups:

1. The no radiation pneumonitis group included patients with mild symptoms equivalent to CTCAE grade 0-1 and with no, patchy or increased density on imaging equivalent to EORTC (LENT-SOMA) grade 2-3.

2. The radiation pneumonitis group included patients with symptoms equivalent to CTCAE grade 2-5 and with patchy or increased density on imaging equivalent to EORTC (LENT-SOMA) grade 2-3. CTCAE grade 2 represents the need for some medical intervention (e.g., steroids), and grade 3 indicates the use of supplemental oxygen (35). None of the patients in this study had CTCAE grade 4 (life-threatening respiratory dysfunction) or 5 (death).

All CT scans were evaluated by an experienced thoracic radiologist focusing on RP.

### Statistics

Patient characteristics were compared by using Student’s *t* test or the chi-square test for continuous and categorical variables, respectively (**Table 2**). We did not have samples from all patients at all time points to analyze the temporal profile of the markers; therefore, we used a univariate general linear model. Markers were categorized as 0: baseline, before radiotherapy; 1: last day of radiotherapy; 2: 1-1.5 months after radiotherapy; and 3, 6, 9 and 12 months after radiotherapy. Markers were log-transformed due to a skewed distribution. Markers were used as dependent variables, RP (yes/no) and time were used as fixed factors, and their interaction (RP*time) and patient number were used as random factors. To limit multiple comparisons, *post hoc* testing was performed only on variables where RP (between groups) or the RP*time interaction (paired comparisons) was significant. This model was also used within the two radiotherapy groups. When comparing levels between RP groups, MANCOVA was used for each time point with smoking, radiotherapy, and stage as covariates. Paired differences were assessed with paired t tests.

**Table 2:** Patient characteristics in relation to study outcomes.

	Radiation Pneumonitis		2-year mortality		5-year mortality	
	No (n = 47)	Yes (n = 19)	No (n = 46)	Yes (n = 20)	No (n = 25)	Yes (n = 41)
Age, years	73.7 (6.8)	71.2 (9.4)	73.4 (7.3)	71.9 (8.4)	73.0 (6.7)	72.9 (8.2)
Male sex	25 (53%)	9 (47%)	20 (44%)	14 (70%)*	13 (52%)	21 (51%)
SBRT	36 (77%)**	8 (42%)	34 (74%)	10 (50%)	20 (80%)	24 (59%)
CCRT	11 (23%)	11 (58%)*	12 (26%)	10 (50%)	5 (20%)	17 (42%)
Previous smoker	36 (77%)**	8 (42%)	34 (74%)	10 (50%)	18 (72%)	26 (63%)
COPD	27 (57%)	9 (47%)	25 (54%)	11 (55%)	12 (48%)	24 (59%)
Morphology	15 (38%)	7 (41%)	12 (32%)	10 (53%)	8 (33%)	14 (42%)
Stage III	8 (17%)	8 (44%)*	9 (20%)	7 (35%)	4 (16%)	12 (30%)
Emphysema	22 (47%)	9 (47%)	23 (50%)	8 (40%)	11 (44%)	20 (49%)
Radiation Pneumonitis			10 (22%)	9 (45%)	6 (24%)	13 (32%)

CCRT Concurrent chemoradiation therapy; SBRT Stereotactic body radiation therapy.*p<0.05, **p<0.01.

The discriminatory properties of baseline levels of serum markers in relation to 2- and 5-year mortality were assessed by receiver operating characteristic (ROC) analysis. The association between significant markers and mortality was further assessed with Cox regression analysis. Log levels of serum markers were standardized, and hazard ratios (HRs) were expressed as the risk per SD of the marker. As the sample size was limited, the most important baseline characteristics were tested as covariates one by one as well as with a propensity score composed of all of them. The temporal profile of the selected serum markers in relation to mortality was also assessed in the univariate general linear model replacing RP with 2- or 5-year mortality. When comparing levels between survivors and nonsurvivors, MANCOVA was used for each time point with sex as a covariate.

## Results

### Patients

**Figure 1:**
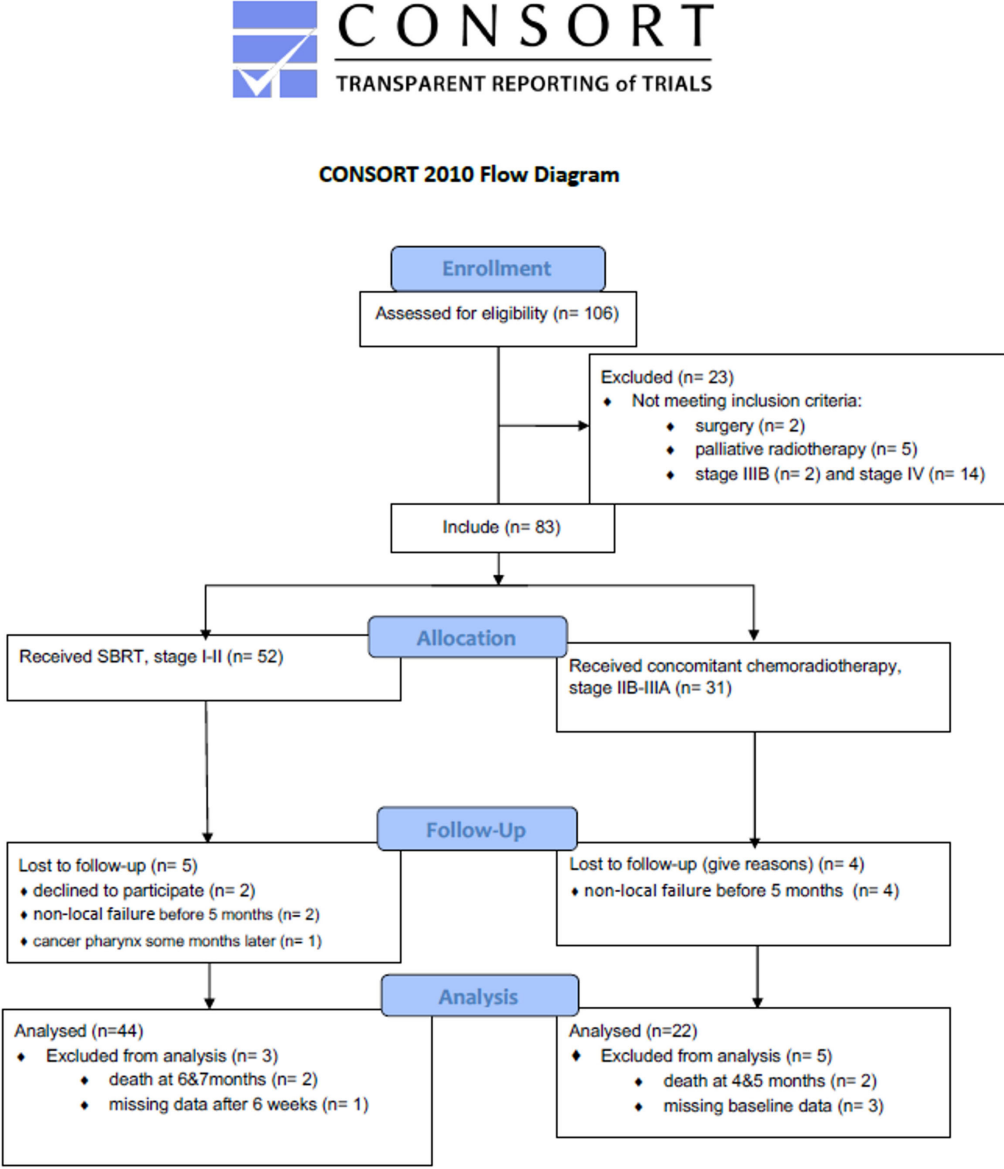
The CONSORT study flow diagram.

The CONSORT study flow diagram is presented in **Figure 1**. The study population and baseline characteristics in relation to the study outcomes are presented in **Table 2**. From February 2014 until December 2017, 66 eligible patients were included with a mean age of 73 years (range 51-90), of which 34 (52%) were male. There were no EGFR-positive patients in our study cohort. ALK fusion testing did not start until 2018 in Norway, so we have no information about this. Nineteen (29%) patients developed RP, which occurred more frequently and earlier in patients receiving CCRT (n=22) than in those receiving SBRT (n=44) and was less frequent in previous smokers and more frequent with advanced stage. RP on CT occurred after a median of 4.9 months after SBRT and 3 months after CCRP. The NSCLC recurrence percentage was similar among patients with and without RP (42% and 40%), the mean observation time was 30 months. Of the patients with RP, 75% had recurrence within 12 months versus 47% in patients without RP. Mortality was assessed at 2 and 5 years, in which 20 (30%) and 41 (62%) patients died, respectively. The only significant difference with regard to these mortality groups was a higher proportion of male patients who died before the 2-year follow-up.

### Serum Markers and RP

**Figure 2:**
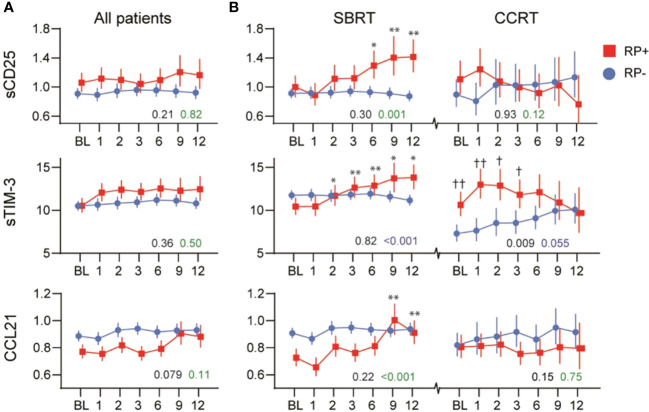
Temporal profile of sCD25, sTIM-3 and CCL21 in relation to radiation pneumonitis (RP) in **(A)** all patients and **(B)** within patients receiving stereotactic body radiation therapy (SBRT) or concurrent chemoradiation therapy (CCRT). The black p-value represents the effect of RP from the univariate general linear model, while the green p-value represents the interaction with time (RP*time). *p < 0.05, **p < 0.01 *vs.* baseline. ^†^p < 0.05, ^††^p < 0.01 vs. RP- same time-point.

The levels of serum markers in relation to RP, measured from baseline until 12 months after treatment, are presented in **Supplementary Table 1**. When evaluating the markers in relation to RP in the group as a whole, no significant difference in levels or temporal profile during the 12-month blood sampling was detected (**Figure 2A** and **Supplementary Figure 1A**). Further evaluation within the radiotherapy groups revealed that in patients receiving SBRT, an increase in sCD25, sTIM-3 and CCL21 levels was observed at the last 6 months of observation time in patients who developed RP (**Figure 2B**). In patients receiving CCRT, those who developed RP were characterized by higher sTIM-3 levels during the first 3 months of follow-up (**Figure 2**). Serum levels of MPO, sPD-1, sCD14, sCD163 and CCL19 showed no significant differences in relation to RP in the two radiation groups (SBRT and CCRT) (**Supplementary Figure 1**).

No difference in the level or course of the markers were found between patients receiving CCRT or conventionally fractionated radiotherapy alone, last group included only 4 patients.

### Serum Markers and Mortality

**Figure 3:**
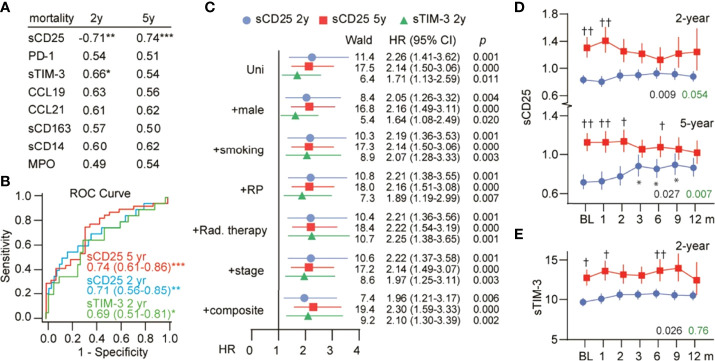
Serum markers and mortality. **(A)** ROC analysis showing the AUC for baseline serum markers in relation to 2- and 5-year mortality. *p < 0.05, **p < 0.01, ***p < 0.001. **(B)** ROC curves for sCD25 and sTIM-3 with AUC (95% CI) in text. **(C)** Cox-regression analysis of baseline sCD25 and sTIM-3 in relation to mortality. HRs represent risk per 1 SD (log) change in serum markers and are shown with inclusion of the strongest baseline predictors as well as a composite score of these. Temporal profile of **(D)** sCD25 and **(E)** sTIM-3 in survivors (blue) and nonsurvivors (red). The black p-value represents the effect of mortality from the univariate general linear model, while the green p-value represents the interaction with time (mortality*time). *p < 0.05 vs. baseline. ^†^p < 0.05, ^††^p < 0.01 *vs.* survivors at the same time point.

We next evaluated whether baseline levels of serum markers could predict 2- and 5-year mortality by discriminatory analysis. As shown in **Figures 3A, B**, sCD25 was associated with both mortality outcomes, while sTIM-3 was associated with 2-year mortality. The areas under the curve (AUCs) and 95% confidence intervals (CIs) for all markers are shown in **Supplementary Table 2**.

We assessed these associations further using survival analysis. As shown in **Figure 3C**, a one SD increase in baseline sCD25 was associated with a 2.3- and 2.1-fold higher risk of death at the 2- and 5-year follow-up, respectively. Furthermore, inclusion of the strongest baseline predictors of mortality (**Supplementary Table 3**) or a composite of these predictors had no influence on the association between sCD25 and mortality. A one SD increase in baseline sTIM-3 was associated with a 1.7-fold higher risk of 2-year mortality and was not influenced by covariates.

Evaluation of the temporal profile of sCD25 and sTIM-3 in relation to these outcomes is shown in **Figures 3D, E**. For sCD25, high levels were observed during the first two months in those who died. sTIM-3 remained mildly elevated in those who died during the 12-month blood sampling.

## Discussion

The present study evaluated serum leukocyte activation markers in NSCLC cancer patients in relation to RP and mortality. Whereas we found no associations with neutrophil and monocyte activation markers, which are traditionally thought to reflect acute and, to some degree, chronic (monocyte) inflammation, we found increased levels and different temporal profiles according to radiation treatment of the T cell activation marker sCD25, T cell exhaustion marker sTIM-3 and chemotactic T cell signal CCL21 in patients who developed RP. Moreover, sTIM-3 and, in particular, sCD25 predicted mortality independent of other demographics, including RP and the mode of radiation treatment. Our study suggests that the development of adverse outcomes in non-small-cell lung cancer patients is linked to T cell activation and exhaustion.

T cell activation is typical in both cancer progression (36), among others, in NSCLC (37, 38) and pneumonitis (39) due to persistent T cell receptor stimulation and is accompanied by the expression of inhibitory receptors such as PD-1 and TIM-3 (40–42) and T cell dysfunction (40). At the same time, radiotherapy modulates several immunological processes: revelation of antigens, activation of T lymphocytes, recruitment and accumulation of T cells in the tumour, and acknowledgement and killing of tumour cells by T lymphocytes. Our finding of increased levels of T cell activation and exhaustion markers in patients who developed RP indicates an active role in RP by likely overstimulation of T cells.

We could not, however, find any previous studies evaluating circulating levels of T cell activation and exhaustion markers in NSCLC or in response to RP. Herein, we found that changes in the levels of T cell markers in relation to RP occurred at different times according to radiation treatment. SBRT delivers the radiation dose to the pulmonary tumour more precisely than CCRT, allowing an escalation of SBRT treatment doses far beyond traditional conventional radiotherapy, damaging healthy lung tissue to a lesser extent and triggering RP more rarely and later than CCRT (11). While T cell exhaustion is mostly used in relation to chronic infections, we speculate that the enhanced early levels of sTIM-3 (i.e., within 3 months) in the CCRT group, without enhanced sCD25 levels, could reflect the effects of more acute RP, which is seen 1-3 months after CCRT (43–46), as a mechanism to prevent persistent and overshooting T cell activation. The more gradual increases in sTIM-3, sCD25 and CCL21 within the SBRT group correlate with the later onset of RP in this group occurring after 5-10 months (9–11, 47–49). Although PD-1, another T cell exhaustion marker, was not significantly associated with RP, the temporal trajectory was similar to sTIM-3 in the SBRT group but not in the CCRT group, possibly reflecting some different effects on T cell subsets of these radiation modalities. The concurrent increase in the T cell chemoattractant CCL21 in the SBRT group could potentially be linked to T cell migration or effects in regional lymph nodes (50).

As T cell exhaustion and dysfunction seem to be hallmarks of cancer progression (51) and are the targets of current immunotherapy (52), it is tempting to speculate that increased sTIM-3 and sCD25 could link RP and poor prognosis in lung cancer. However, while severe RP is associated with poor short-term outcome, prognosis in milder cases seems to be more dependent on underlying factors (53–55). Furthermore, we found that the association between sCD25, sTIM-3 and mortality was independent of both RP and the mode of radiotherapy. In patients with glioblastoma (56) and melanoma (57), only tumour-infiltrating, but not peripheral, T cells showed enhanced levels of exhaustion-associated inhibitory receptors.

Thus, our findings could potentially reflect immunological abnormalities in the TME, which has been associated with poor prognosis in lung cancer (58). This may also support double immunotherapy targeting both TIM-3 and PD-1, which has indeed been shown to improve antitumour T cell responses in preclinical models (59–61). Moreover, inflammation is a recognized hallmark in carcinogenesis (62). Macrophages are associated with chronic inflammation in cancer initiation and promotion and are also involved in tumour progression and metastasis (63). Tumour-associated macrophages correlate with poor prognosis (64). Radiotherapy changes the TME by attacking cancer cells, blood vessels and immune-related cells in the TME. Immunological changes occur in the affected tissue first and later become measurable in peripheral blood. The absence of neutrophil and monocyte activation markers after radiotherapy in our study suggests that those markers do not play a crucial role in the pathogenesis of RP.

We were unable to find studies evaluating serum levels of the monocyte/macrophage activation markers sCD14 or sCD163 or the neutrophil activation marker MPO in relation to survival in lung cancer patients. Enhanced tumour-associated CD163 expression by immunohistochemistry (IHC) was associated with poor survival in a small study (65), while a larger study (n=335) found no association between CD66b(+) neutrophils and CD163(+) macrophages and survival in lung cancer patients (66). Nevertheless, our data suggest that any potential activation of these cells in RP or in relation to disease progression is not reflected by circulating levels of activation markers.

Treatment with immune checkpoint inhibitors (ICIs) is a relatively new class of therapeutic agents that have shown impressive anticancer effects for a number of solid cancer types. ICI-induced pneumonitis is a rare but severe side effect and is occasionally fatal. Life-threatening pneumonitis has been reported in up to 2% of cases (67–69). The incidence of ICI-induced pneumonitis is higher in NSCLC than in other cancer types and in combined therapy with radiotherapy or chemotherapy (6.5%–10%) than in monotherapy (3%–4%) (70, 71). These findings can be useful in the study of ICI-induced pneumonitis pathogenesis.

The occurrence of RP after SBRT is lower than after CCRT (13, 14, 16, 72) and severe RP is quite uncommon (7, 8, 73). SBRT techniques allow minimizes the size of the planning target volume (PTV) and meaning the normal lung tissue in the target volume. The larger irradiation fields increase values of inflammation markers. In the present study, however, too few patients underwent conventionally fractioned radiotherapy alone in the CCRT group to make any reliable comparison.

In conclusion, our findings showing increased sCD25 and sTIM-3 in relation to RP and survival suggest that persistent T cell activation and exhaustion may contribute to progression and adverse outcomes in locally advanced NSCLC and support T cell-targeted treatment in this disorder.

## Data Availability Statement

The raw data supporting the conclusions of this article will be made available by the authors, without undue reservation.

## Ethics Statement

The studies involving human participants were reviewed and approved by Regional Ethical Committee, REK nr. 2013/169/REK sør-øst D. The patients/participants provided their written informed consent to participate in this study.

## Author Contributions

Conception and design: JB, TU, PA, ÅH. Provision of study materials or patients: JB, M-BB, ÅH. Collection and assembly of data: JB, TU. Data analysis and interpretation: JB, TU, PA, ÅH, ARH, ML. Manuscript writing: JB, TU, PA, ÅH. Final approval of manuscript: All authors.

## Funding

This research was funded by the Regional Health Authorities in Southeast Norway (grant 2015058), the Vestfold Hospital Trust (grant 197430) and an unrestricted grant from Boehringer Ingelheim Norway (grant 197430).

## Conflict of Interest

The authors declare that the research was conducted in the absence of any commercial or financial relationships that could be construed as a potential conflict of interest.

## Publisher’s Note

All claims expressed in this article are solely those of the authors and do not necessarily represent those of their affiliated organizations, or those of the publisher, the editors and the reviewers. Any product that may be evaluated in this article, or claim that may be made by its manufacturer, is not guaranteed or endorsed by the publisher.

## References

[1] Sung H Ferlay J Siegel RL Laversanne M Soerjomataram I Jemal A. Global Cancer Statistics 2020: GLOBOCAN Estimates of Incidence and Mortality Worldwide for 36 Cancers in 185 Countries. CA: A Cancer J Clin (2021) 71(3):209–49. doi: 10.3322/caac.21660 33538338

[2] Sorokin S. The Cells of the Lung. In: Morphology of the Experimental Respiratory Carcinogenesis AEC Symposium Series 21 Oak Ridge, Tennessee: U.S. Atomic energy commission, Division of technical information (1970). p. 3–43.

[3] Phillips TL. An Ultrastructural Study of the Development of Radiation Injury in the Lung. Radiology (1966) 87(1):49–54. doi: 10.1148/87.1.49 5940475

[4] Rubin P Casarett GW. Clinical Radiation Pathologyplos Volume I (1968). Available at: https://www.osti.gov/biblio/4798563-clinical-radiation-pathology-volume.

[5] Coggle JE Lambert BE Moores SR. Radiation Effects in the Lung. Environ Health Perspect (1986) 70:261–91. doi: 10.1289/ehp.8670261 PMC1474274 3549278

[6] Stephans KL Djemil T Reddy CA Gajdos SM Kolar M Machuzak M. Comprehensive Analysis of Pulmonary Function Test (PFT) Changes After Stereotactic Body Radiotherapy (SBRT) for Stage I Lung Cancer in Medically Inoperable Patients. J Thorac Oncol (2009) 4(7):838–44. doi: 10.1097/JTO.0b013e3181a99ff6 19487961

[7] Stone B Mangona VS Johnson MD Ye H Grills IS. Changes in Pulmonary Function Following Image-Guided Stereotactic Lung Radiotherapy: Neither Lower Baseline Nor Post-SBRT Pulmonary Function Are Associated With Worse Overall Survival. J Thorac Oncol (2015) 10(12):1762–9. doi: 10.1097/JTO.0000000000000670 26334751

[8] Ferrero C Badellino S Filippi AR Focaraccio L Giaj Levra M Levis M. Pulmonary Function and Quality of Life After VMAT-Based Stereotactic Ablative Radiotherapy for Early Stage Inoperable NSCLC: A Prospective Study. Lung Cancer (2015) 89(3):350–6. doi: 10.1016/j.lungcan.2015.06.019 26164208

[9] Kimura T Matsuura K Murakami Y Hashimoto Y Kenjo M Kaneyasu Y. CT Appearance of Radiation Injury of the Lung and Clinical Symptoms After Stereotactic Body Radiation Therapy (SBRT) for Lung Cancers: Are Patients With Pulmonary Emphysema Also Candidates for SBRT for Lung Cancers? Int J Radiat Oncol Biol Phys (2006) 66(2):483–91. doi: 10.1016/j.ijrobp.2006.05.008 16904838

[10] Berg J Ramberg C Haugstvedt JOS Bengtson MB Gabrielsen AM Brustugun OT. Lung Function After Stereotactic Body Radiation Therapy for Early-Stage Non-Small Cell Lung Cancer, Changes and Predictive Markers. Front Oncol (2021) 11:1829. doi: 10.3389/fonc.2021.674731 PMC8181743 34109123

[11] Guckenberger M Heilman K Wulf J Mueller G Beckmann G Flentje M. Pulmonary Injury and Tumor Response After Stereotactic Body Radiotherapy (SBRT): Results of a Serial Follow-Up CT Study. Radiother Oncol (2007) 85(3):435–42. doi: 10.1016/j.radonc.2007.10.044 18053602

[12] Bradley JD Paulus R Komaki R Masters G Blumenschein G Schild S. Standard-Dose Versus High-Dose Conformal Radiotherapy With Concurrent and Consolidation Carboplatin Plus Paclitaxel With or Without Cetuximab for Patients With Stage IIIA or IIIB non-Small-Cell Lung Cancer (RTOG 0617): A Randomised, Two-by-Two Factorial Phase 3 Study. Lancet Oncol (2015) 16(2):187–99. doi: 10.1016/S1470-2045(14)71207-0 PMC4419359 25601342

[13] Park YH Kim JS. Predictors of Radiation Pneumonitis and Pulmonary Function Changes After Concurrent Chemoradiotherapy of non-Small Cell Lung Cancer. Radiat Oncol J (2013) 31(1):34–40. doi: 10.3857/roj.2013.31.1.34 23620867 PMC3633229

[14] Tsujino K Hirota S Endo M Obayashi K Kotani Y Satouchi M. Predictive Value of Dose-Volume Histogram Parameters for Predicting Radiation Pneumonitis After Concurrent Chemoradiation for Lung Cancer. Int J Radiat Oncol Biol Phys (2003) 55(1):110–5. doi: 10.1016/S0360-3016(02)03807-5 12504042

[15] Wang J Cao J Yuan S Ji W Arenberg D Dai J. Poor Baseline Pulmonary Function may Not Increase the Risk of Radiation-Induced Lung Toxicity. Int J Radiat Oncol Biol Phys (2013) 85(3):798–804. doi: 10.1016/j.ijrobp.2012.06.040 22836048 PMC3646086

[16] Palma DA Senan S Tsujino K Barriger RB Rengan R Moreno M. Predicting Radiation Pneumonitis After Chemoradiation Therapy for Lung Cancer: An International Individual Patient Data Meta-Analysis. Int J Radiat Oncol Biol Phys (2013) 85(2):444–50. doi: 10.1016/j.ijrobp.2012.04.043 PMC3448004 22682812

[17] O’Rourke N Figuls MR Bernadó NF Macbeth F. Concurrent Chemoradiotherapy in non-Small Cell Lung Cancer. Cochrane Database Syst Rev (2010) 6):CD002140. doi: 10.1002/14651858.CD002140.pub3/full PMC12584098 20556756

[18] Hanahan D Coussens LM. Accessories to the Crime: Functions of Cells Recruited to the Tumor Microenvironment. Cancer Cell (2012) 21(3):309–22. doi: 10.1016/j.ccr.2012.02.022 22439926

[19] Sadeghi Rad H Monkman J Warkiani ME Ladwa R O’Byrne K Rezaei N. Understanding the Tumor Microenvironment for Effective Immunotherapy. Med Res Rev (2021) 41(3):1474–98. doi: 10.1002/med.21765 PMC8247330 33277742

[20] Kim SR Kim DI Kim SH Lee H Lee KS Cho SH. NLRP3 Inflammasome Activation by Mitochondrial ROS in Bronchial Epithelial Cells is Required for Allergic Inflammation. Cell Death Dis (2014) 5:e1498. doi: 10.1038/cddis.2014.460 25356867 PMC4237270

[21] Liu X Chen Z. The Pathophysiological Role of Mitochondrial Oxidative Stress in Lung Diseases. J Trans Med (2017) 15(1):207. doi: 10.1186/s12967-017-1306-5 PMC5640915 29029603

[22] Ghafoori P Marks LB Vujaskovic Z Kelsey CR. Radiation-Induced Lung Injury. Assessment, Management, and Prevention. Oncol (Williston Park) (2008) 22(1):37–47. 18251282

[23] Darby IA Hewitson TD. Fibroblast Differentiation in Wound Healing and Fibrosis. Int Rev Cytol (2007) 257:143–79. doi: 10.1016/S0074-7696(07)57004-X 17280897

[24] Barker HE Paget JTE Khan AA Harrington KJ. The Tumour Microenvironment After Radiotherapy: Mechanisms of Resistance and Recurrence. Nat Rev Cancer (2015) 15(7):409–25. doi: 10.1038/nrc3958 PMC4896389 26105538

[25] Tsoutsou PG Belkacemi Y Gligorov J Kuten A Boussen H Bese N. Optimal Sequence of Implied Modalities in the Adjuvant Setting of Breast Cancer Treatment: An Update on Issues to Consider. Oncologist (2010) 15(11):1169–78. doi: 10.1634/theoncologist.2010-0187 PMC3227907 21041378

[26] Barthelemy-Brichant N Bosquée L Cataldo D Corhay JL Gustin M Seidel L. Increased IL-6 and TGF-Beta1 Concentrations in Bronchoalveolar Lavage Fluid Associated With Thoracic Radiotherapy. Int J Radiat Oncol Biol Phys (2004) 58(3):758–67. doi: 10.1016/S0360-3016(03)01614-6 14967431

[27] Ryter SW Kim HP Hoetzel A Park JW Nakahira K Wang X. Mechanisms of Cell Death in Oxidative Stress. Antioxid Redox Signal (2007) 9(1):49–89. doi: 10.1089/ars.2007.9.49 17115887

[28] Zhang B Wang Y Pang X Su Y Ai G Wang T. ER Stress Induced by Ionising Radiation in IEC-6 Cells. Int J Radiat Biol (2010) 86(6):429–35. doi: 10.3109/09553001003668014 20470193

[29] Tan Z Xue H Sun Y Zhang C Song Y Qi Y. The Role of Tumor Inflammatory Microenvironment in Lung Cancer. Front Pharmacol (2021) 12. doi: 10.3389/fphar.2021.688625 PMC8166205 34079469

[30] O’Callaghan DS O’Donnell D O’Connell F O’Byrne KJ. The Role of Inflammation in the Pathogenesis of non-Small Cell Lung Cancer. J Thorac Oncol (2010) 5(12):2024–36. doi: 10.1097/JTO.0b013e3181f387e4 21155185

[31] Siva S MacManus M Kron T Best N Smith J Lobachevsky P. A Pattern of Early Radiation-Induced Inflammatory Cytokine Expression is Associated With Lung Toxicity in Patients With non-Small Cell Lung Cancer. PLoS One (2014) 9(10):e109560. doi: 10.1371/journal.pone.0109560 25289758 PMC4188745

[32] Lierova A Jelicova M Nemcova M Proksova M Pejchal J Zarybnicka L. Cytokines and Radiation-Induced Pulmonary Injuries. J Radiat Res (2018) 59(6):709–53. doi: 10.1093/jrr/rry067 PMC6251431 30169853

[33] Kong FM Ao X Wang L Lawrence TS. The Use of Blood Biomarkers to Predict Radiation Lung Toxicity: A Potential Strategy to Individualize Thoracic Radiation Therapy. Cancer Control (2008) 15(2):140–50. doi: 10.1177/107327480801500206 18376381

[34] Giuranno L Ient J De Ruysscher D Vooijs MA. Radiation-Induced Lung Injury (RILI). Front Oncol (2019) 9:877. doi: 10.3389/fonc.2019.00877 31555602 PMC6743286

[35] Jain V Berman AT. Radiation Pneumonitis: Old Problem, New Tricks. Cancers (Basel) (2018) 10(7):222. doi: 10.3390/cancers10070222 PMC6071030 29970850

[36] Wherry EJ Kurachi M. Molecular and Cellular Insights Into T Cell Exhaustion. Nat Rev Immunol (2015) 15(8):486–99. doi: 10.1038/nri3862 PMC4889009 26205583

[37] van der Leun AM Thommen DS Schumacher TN. CD8+ T Cell States in Human Cancer: Insights From Single-Cell Analysis. Nat Rev Cancer (2020) 20(4):218–32. doi: 10.1038/s41568-019-0235-4 PMC7115982 32024970

[38] Nguyen LT Ohashi PS. Clinical Blockade of PD1 and LAG3 — Potential Mechanisms of Action. Nat Rev Immunol (2015) 15(1):45–56. doi: 10.1038/nri3790 25534622

[39] Song D Yan F Fu H Li L Hao J Zhu Z. A Cellular Census of Human Peripheral Immune Cells Identifies Novel Cell States in Lung Diseases. Clin Transl Med (2021) 11(11):e579. doi: 10.1002/ctm2.579 34841705 PMC8611783

[40] Thommen DS Schreiner J Müller P Herzig P Roller A Belousov A. Progression of Lung Cancer Is Associated With Increased Dysfunction of T Cells Defined by Coexpression of Multiple Inhibitory Receptors. Cancer Immunol Res (2015) 3(12):1344–55. doi: 10.1158/2326-6066.CIR-15-0097 26253731

[41] Anderson AC. Tim-3: An Emerging Target in the Cancer Immunotherapy Landscape. Cancer Immunol Res (2014) 2(5):393–8. doi: 10.1158/2326-6066.CIR-14-0039 24795351

[42] Fourcade J Sun Z Benallaoua M Guillaume P Luescher IF Sander C. Upregulation of Tim-3 and PD-1 Expression is Associated With Tumor Antigen-Specific CD8+ T Cell Dysfunction in Melanoma Patients. J Exp Med (2010) 207(10):2175–86. doi: 10.1084/jem.20100637 PMC2947081 20819923

[43] Wirsdörfer F Jendrossek V. The Role of Lymphocytes in Radiotherapy-Induced Adverse Late Effects in the Lung. Front Immunol (2016) 7:591. doi: 10.3389/fimmu.2016.00591 28018357 PMC5155013

[44] Hanania AN Mainwaring W Ghebre YT Hanania NA Ludwig M. Radiation-Induced Lung Injury: Assessment and Management. Chest (2019) 156(1):150–62. doi: 10.1016/j.chest.2019.03.033 PMC8097634 30998908

[45] Gross NJ. Pulmonary Effects of Radiation Therapy. Ann Intern Med (1977) 86(1):81–92. doi: 10.7326/0003-4819-86-1-81 319723

[46] Abratt RP Morgan GW Silvestri G Willcox P. Pulmonary Complications of Radiation Therapy. Clin Chest Med (2004) 25(1):167–77. doi: 10.1016/S0272-5231(03)00126-6 15062608

[47] Bradley J. Radiographic Response and Clinical Toxicity Following SBRT for Stage I Lung Cancer. J Thorac Oncol (2007) 2(7, Supplement 3):S118–24. doi: 10.1097/JTO.0b013e318074e50c 17603307

[48] Saha A Beasley M Hatton N Dickinson P Franks K Clarke K. Clinical and Dosimetric Predictors of Radiation Pneumonitis in Early-Stage Lung Cancer Treated With Stereotactic Ablative Radiotherapy (SABR) - An Analysis of UK’s Largest Cohort of Lung SABR Patients. Radiother Oncol (2021) 156:153–9. doi: 10.1016/j.radonc.2020.12.015 33333139

[49] Timmerman R McGarry R Yiannoutsos C Papiez L Tudor K DeLuca J. Excessive Toxicity When Treating Central Tumors in a Phase II Study of Stereotactic Body Radiation Therapy for Medically Inoperable Early-Stage Lung Cancer. J Clin Oncol (2006) 24(30):4833–9. doi: 10.1200/JCO.2006.07.5937 17050868

[50] Sharma S Kadam P Dubinett S. CCL21 Programs Immune Activity in Tumor Microenvironment. Adv Exp Med Biol (2020) 1231:67–78. doi: 10.1007/978-3-030-36667-4_7 32060847

[51] Zhang Z Liu S Zhang B Qiao L Zhang Y Zhang Y. T Cell Dysfunction and Exhaustion in Cancer. Front Cell Dev Biol (2020) 8:17. doi: 10.3389/fcell.2020.00017 32117960 PMC7027373

[52] Pauken KE Wherry EJ. Overcoming T Cell Exhaustion in Infection and Cancer. Trends Immunol (2015) 36(4):265–76. doi: 10.1016/j.it.2015.02.008 PMC4393798 25797516

[53] Inoue A Kunitoh H Sekine I Sumi M Tokuuye K Saijo N. Radiation Pneumonitis in Lung Cancer Patients: A Retrospective Study of Risk Factors and the Long-Term Prognosis. Int J Radiat Oncol Biol Phys (2001) 49(3):649–55. doi: 10.1016/S0360-3016(00)00783-5 11172945

[54] Keffer S Guy CL Weiss E. Fatal Radiation Pneumonitis: Literature Review and Case Series. Adv Radiat Oncol (2020) 5(2):238–49. doi: 10.1016/j.adro.2019.08.010 PMC7136627 32280824

[55] Onishi H Marino K Yamashita H Terahara A Onimaru R Kokubo M. Case Series of 23 Patients Who Developed Fatal Radiation Pneumonitis After Stereotactic Body Radiotherapy for Lung Cancer. Technol Cancer Res Treat (2018) 17:1533033818801323. doi: 10.1177/1533033818801323 30286697 PMC6174642

[56] Woroniecka K Chongsathidkiet P Rhodin K Kemeny H Dechant C Farber SH. T-Cell Exhaustion Signatures Vary With Tumor Type and Are Severe in Glioblastoma. Clin Cancer Res (2018) 24(17):4175–86. doi: 10.1158/1078-0432.CCR-17-1846 PMC6081269 29437767

[57] Utzschneider DT Legat A Fuertes Marraco SA Carrié L Luescher I Speiser DE. T Cells Maintain an Exhausted Phenotype After Antigen Withdrawal and Population Reexpansion. Nat Immunol (2013) 14(6):603–10. doi: 10.1038/ni.2606 23644506

[58] Jiang W He Y He W Wu G Zhou X Sheng Q. Exhausted Cd8+T Cells in the Tumor Immune Microenvironment: New Pathways to Therapy. Front Immunol (2020) 11. doi: 10.3389/fimmu.2020.622509 PMC7902023 33633741

[59] Acharya N Sabatos-Peyton C Anderson AC. Tim-3 Finds its Place in the Cancer Immunotherapy Landscape. J Immunother Cancer (2020) 8(1):e000911. doi: 10.1136/jitc-2020-000911 32601081 PMC7326247

[60] De Mello RA Zhu JH Iavelberg J Potim AH Simonetti D Silva JA Jr. Current and Future Aspects of TIM-3 as Biomarker or as Potential Targeted in non-Small Cell Lung Cancer Scope: Is There a Role in Clinical Practice? Transl Lung Cancer Res (2020) 9(6):2311–14. doi: 10.21037/tlcr-20-625 PMC7815370 33489794

[61] Friedlaender A Addeo A Banna G. New Emerging Targets in Cancer Immunotherapy: The Role of TIM3. ESMO Open (2019) 4(Suppl 3):e000497. doi: 10.1136/esmoopen-2019-000497 31275616 PMC6579568

[62] Hanahan D Weinberg RA. Hallmarks of Cancer: The Next Generation. Cell (2011) 144(5):646–74. doi: 10.1016/j.cell.2011.02.013 21376230

[63] Condeelis J Pollard JW. Macrophages: Obligate Partners for Tumor Cell Migration, Invasion, and Metastasis. Cell (2006) 124(2):263–6. doi: 10.1016/j.cell.2006.01.007 16439202

[64] Bingle L Brown NJ Lewis CE. The Role of Tumour-Associated Macrophages in Tumour Progression: Implications for New Anticancer Therapies. J Pathol (2002) 196(3):254–65. doi: 10.1002/path.1027 11857487

[65] Lin MW Yang CY Kuo SW Wu CT Chang YL Yang PC. The Prognostic Significance of Pstat1 and CD163 Expressions in Surgically Resected Stage 1 Pulmonary Squamous Cell Carcinomas. Ann Surg Oncol (2016) 23(9):3071–81. doi: 10.1245/s10434-016-5244-x 27150441

[66] Carus A Ladekarl M Hager H Pilegaard H Nielsen PS Donskov F. Tumor-Associated Neutrophils and Macrophages in non-Small Cell Lung Cancer: No Immediate Impact on Patient Outcome. Lung Cancer (2013) 81(1):130–7. doi: 10.1016/j.lungcan.2013.03.003 23540719

[67] Haanen JBAG Carbonnel F Robert C Kerr KM Peters S Larkin J. Management of Toxicities From Immunotherapy: ESMO Clinical Practice Guidelines for Diagnosis, Treatment and Follow-Up. Ann Oncol (2017) 28(suppl_4):iv119–42. doi: 10.1093/annonc/mdx225 28881921

[68] Xing P Zhang F Wang G Xu Y Li C Wang S. Incidence Rates of Immune-Related Adverse Events and Their Correlation With Response in Advanced Solid Tumours Treated With NIVO or NIVO+IPI: A Systematic Review and Meta-Analysis. J Immunother Cancer (2019) 7(1):341. doi: 10.1186/s40425-019-0779-6 31801636 PMC6894272

[69] Brahmer JR Lacchetti C Schneider BJ Atkins MB Brassil KJ Caterino JM. Management of Immune-Related Adverse Events in Patients Treated With Immune Checkpoint Inhibitor Therapy: American Society of Clinical Oncology Clinical Practice Guideline. J Clin Oncol (2018) 36(17):1714–68. doi: 10.1200/JCO.2017.77.6385 PMC6481621 29442540

[70] Naidoo J Wang X Woo KM Iyriboz T Halpenny D Cunningham J. Pneumonitis in Patients Treated With Anti-Programmed Death-1/Programmed Death Ligand 1 Therapy. J Clin Oncol (2017) 35(7):709–17. doi: 10.1200/JCO.2016.68.2005 PMC5559901 27646942

[71] Nishino M Giobbie-Hurder A Hatabu H Ramaiya NH Hodi FS. Incidence of Programmed Cell Death 1 Inhibitor-Related Pneumonitis in Patients With Advanced Cancer: A Systematic Review and Meta-Analysis. JAMA Oncol (2016) 2(12):1607–16. doi: 10.1001/jamaoncol.2016.2453 27540850

[72] Yorke ED Jackson A Rosenzweig KE Merrick SA Gabrys D Venkatraman ES. Dose-Volume Factors Contributing to the Incidence of Radiation Pneumonitis in non-Small-Cell Lung Cancer Patients Treated With Three-Dimensional Conformal Radiation Therapy. Int J Radiat Oncol Biol Phys (2002) 54(2):329–39. doi: 10.1016/S0360-3016(02)02929-2 12243805

[73] Videtic GMM Donington J Giuliani M Heinzerling J Karas TZ Kelsey CR. Stereotactic Body Radiation Therapy for Early-Stage non-Small Cell Lung Cancer: Executive Summary of an ASTRO Evidence-Based Guideline. Pract Radiat Oncol (2017) 7(5):295–301. doi: 10.1016/j.prro.2017.04.014 28596092

