# Corrigendum: Circulating T cell activation and exhaustion markers are associated with radiation pneumonitis and poor survival in non-small-cell lung cancer

**DOI:** 10.3389/fimmu.2022.1051156

**Published:** 2022-10-03

**Authors:** Janna Berg, Ann Rita Halvorsen, May-Bente Bengtson, Morten Lindberg, Bente Halvorsen, Pål Aukrust, Åslaug Helland, Thor Ueland

**Affiliations:** ^1^ Department of Medicine, Vestfold Hospital Trust, Tønsberg, Norway; ^2^ Department of Cancer Genetics, Institute for Cancer Research, Norwegian Radium Hospital, Oslo University Hospital, Oslo, Norway; ^3^ Department of Clinical Medicine, University of Oslo, Oslo, Norway; ^4^ Department of Oncology, Oslo University Hospital, Oslo, Norway; ^5^ Department of Medical Biochemistry, Vestfold Hospital Trust, Tønsberg, Norway; ^6^ Research Institute of Internal Medicine, Oslo University Hospital Rikshospitalet, Oslo, Norway; ^7^ Institute of Clinical Medicine, University of Oslo, Oslo, Norway; ^8^ Section of Clinical Immunology and Infectious Diseases, Oslo University Hospital Rikshospitalet, Oslo, Norway; ^9^ K.G. Jebsen Thrombosis Research and Expertise Center, University of Tromsø, Tromsø, Norway

**Keywords:** lung cancer, radiotherapy, stereotactic body radiation therapy, radiation pneumonitis, radiation-induced lung injury (RILI), blood biomarkers, t cell, leukocyte subsets

## Additional affiliation(s)

In the published article, there was an error regarding the affiliation for Janna Berg. As well as having affiliation(s) 1,2, she should also have “3. Department of Clinical Medicine, University of Oslo, Oslo, Norway.”

The authors apologize for this error and state that this does not change the scientific conclusions of the article in any way. The original article has been updated.

## Publisher’s note

All claims expressed in this article are solely those of the authors and do not necessarily represent those of their affiliated organizations, or those of the publisher, the editors and the reviewers. Any product that may be evaluated in this article, or claim that may be made by its manufacturer, is not guaranteed or endorsed by the publisher.

