# Supplementary material for: Circulating T Cell Activation and Exhaustion Markers Are Associated With Radiation Pneumonitis and Poor Survival in Non-Small-Cell Lung Cancer
Source: Front Immunol. 2022 Jul 14;13:875152. doi: 10.3389/fimmu.2022.875152 (PMC9329944; doi:10.3389/fimmu.2022.875152)

# Supplementary Figures and Tables

**Supplemental Table 1.** Plasma leukocyte activation markers during temporal sampling in relation to Radiation Pneumonitis (RP) according to radiation therapy

|  | **Baseline** (n=19/47) | **Last radiation day** (n=16/32) | **1-1.5 month** (n=16/31) | **3 month** (n=17/39) | **6 month** (n=15/36) | **9 month** (n=10/34) | **12 month** (10/35) |
| --- | --- | --- | --- | --- | --- | --- | --- |
| **sTIM-3** |  |  |  |  |  |  |  |
| RP | 11.2 (9.4, 13.1) | 13.0 (10.7, 15.3) | 13.9 (10.6, 17.2) | 13.3 (10.0-16.6) | 13.6 (11.3, 15.8) | 13.1 (10.8, 15.4) | 12.5 (8.9, 16.2) |
| non-RP | 12.0 (10.0-14.1) | 12.9 (10.6, 15.3) | 13.1 (10.6, 15.6) | 12.0 (10.2, 13.7) | 13.2 (10.8, 15.5) | 12.4 (10.4, 14.5) | 10.8 (9.3, 12.2) |
| **PD-1** |  |  |  |  |  |  |  |
| RP | 469 (259, 678) | 380 (191, 568) | 588 (281, 895) | 382 (262, 503) | 470 (283, 656) | 429 (235, 622) | 503 (331, 675) |
| non-RP | 404 (300, 508) | 385 (256, 512) | 360 (268, 452) | 384 (285, 482) | 404 (309, 500) | 387 (293, 480) | 345 (268, 421) |
| **sCD25** |  |  |  |  |  |  |  |
| RP | 1.35 (0.79, 1.92) | 1.39 (0.90, 1.87) | 1.54 (0.76, 2.32) | 1.39 (0.80, 1.99) | 1.29 (0.95, 1.63) | 1.30 (0.74-1.85) | 1.17 (0.66, 1.67) |
| non-RP | 1.05 (0.87, 1.23) | 1.04 (0.83, 1.24) | 1.08 (0.85, 1.32) | 1.07 (0.87, 1.26) | 1.14 (0.91, 1.37) | 1.04 (0.85, 1.23) | 0.92 (0.76, 1.07) |
| **CCL21** |  |  |  |  |  |  |  |
| RP | 0.80 (0.71, 0.88) | 0.81 (0.64, 0.98) | 0.84 (0.73, 0.96) | 0.77 (0.69, 0.85) | 0.83 (0.73, 0.92) | 0.95 (0.80, 1.10) | 0.90 (0.79, 1.01) |
| non-RP | 0.93 (0.84, 1.03) | 0.89 (0.79, 0.99) | 0.93 (0.83, 1.04) | 0.98 (0.87, 1.09) | 0.95 (0.86, 1.03) | 0.95 (0.85, 1.04) | 0.90 (0.83, 0.97) |
| **CCL19** |  |  |  |  |  |  |  |
| RP | 254 (193, 315) | 241 (167, 315) | 259 (183, 336) | 208 (140, 276) | 239 (150, 327) | 264 (156, 372) | 299 (205, 393) |
| non-RP | 241 (194, 289) | 251 (196, 306) | 206 (169, 242) | 233 (1.95, 2.72) | 239 (194, 283) | 239 (197, 281) | 213 (173, 253) |
| **MPO** |  |  |  |  |  |  |  |
| RP | 145 (112, 178) | 182 (144, 220) | 157 (121, 194) | 161 (116, 207) | 139 (110, 168) | 163 (120, 206) | 150 (113, 187) |
| non-RP | 144 (125, 162) | 178 (147, 209) | 142 (120, 164) | 143 (121, 165) | 133 (109, 156) | 146 (123, 169) | 126 (106, 146) |
| **sCD163** |  |  |  |  |  |  |  |
| RP | 1066 (834, 1299) | 1050 (768, 1332) | 1214 (898, 530) | 1045 (742, 1349) | 1076 (754, 1399) | 1022 (751, 1293) | 1309 (855, 1763) |
| non-RP | 811 (709, 913) | 735 (649, 820) | 913 (774, 1053) | 881 (780, 983) | 960 (834, 1085) | 940 (788, 1092) | 818 (699, 937) |
| **sCD14** |  |  |  |  |  |  |  |
| RP | 1.45 (1.28, 1.62) | 1.49 (1.34, 1.64) | 1.62 (1.37, 1.86) | 1.41 (1.25, 1.56) | 1.53 (1.30, 1.76) | 1.48 (1.20, 1.75) | 1.31 (1.07, 1.54) |
| non-RP | 1.34 (1.23, 1.45) | 1.29 (1.14, 1.45) | 1.30 (1.18, 1.41) | 1.37 (1.25, 149) | 1.33 (1.18, 1.47) | 1.39 (1.25, 1.53) | 1.30 (1.16, 1.43) |

**Supplemental Table 2.** ROC analysis of baseline plasma markers in relation to

2- and 5-year mortality

|  | 2-year mortality | | 5-year mortality | |
| --- | --- | --- | --- | --- |
|  | AUC (95% CI) | *p* | AUC (95% CI) | *p* |
| sCD25 | 0.71 (0.56-0.85) | 0.007 | 0.74 (0.61-0.86) | <0.001 |
| PD-1 | 0.54 (0.38-0.71) | 0.62 | 0.51 (0.37-0.65) | 0.861 |
| sTIM3 | 0.66 (0.51-0.81) | 0.040 | 0.54 (0.39-0.69) | 0.574 |
| CCL19 | 0.63 (0.48-0.78) | 0.097 | 0.56 (0.41-0.70) | 0.444 |
| CCL21 | 0.61 (0.45-0.77) | 0.17 | 0.62 (0.48-0.75) | 0.104 |
| sCD163 | 0.57 (0.40-0.73) | 0.43 | 0.50 (0.35-0.64) | 0.957 |
| sCD14 | 0.60 (0.44-0.76) | 0.21 | 0.62 (0.48-0.77) | 0.083 |
| MPO | 0.49 (0.33-0.64) | 0.87 | 0.54 (0.40-0.68) | 0.557 |

**Supplemental Table 3.** Univariate cox-regression analysis of patient characteristics in relation to 2- and 5-year mortality

|  | 2-year mortality | | | 5-year mortality | | |
| --- | --- | --- | --- | --- | --- | --- |
|  | Wald | HR (95% CI) | *p* | Wald | HR (95% CI) | *p* |
| Age | 0.4 | 0.98 (0.93-1.04) | 0.525 | 0.1 | 0.99 (0.95-1.04) | 0.775 |
| Male sex | 3.9 | 0.38 (0.15-0.99) | 0.048 | 0.3 | 0.83 (0.45-1.54) | 0.560 |
| Smoking | 3.7 | 0.42 (0.18-1.02) | 0.055 | 2.5 | 0.6 (0.32-1.14) | 0.116 |
| Radiation pneumonia | 3.6 | 2.34 (0.97-5.66) | 0.058 | 2.2 | 1.65 (0.85-3.19) | 0.140 |
| CCRT | 3.7 | 2.36 (0.98-5.68) | 0.055 | 6.3 | 2.25 (1.2-4.23) | 0.012 |
| Stage III/IV | 1.8 | 1.89 (0.75-4.74) | 0.176 | 3.0 | 1.82 (0.92-3.59) | 0.083 |
| Lymphocyte count | 0.1 | 0.94 (0.59-1.47) | 0.775 | 0.1 | 0.94 (0.68-1.31) | 0.732 |
| COPD | 0.0 | 0.97 (0.4-2.33) | 0.936 | 0.2 | 1.14 (0.61-2.13) | 0.671 |

**Supplemental Figure 1.** Temporal profile of plasma markers in relation to radiation pneumonitis (RP) in A) all patients and B) within patients receiving stereotactic body radiation therapy (SBRT) or concurrent chemoradiation therapy (CCRT). Black p-value represents the effect of RP from the univariate general linear model while the green p-value represents the interaction with time (RP*time).


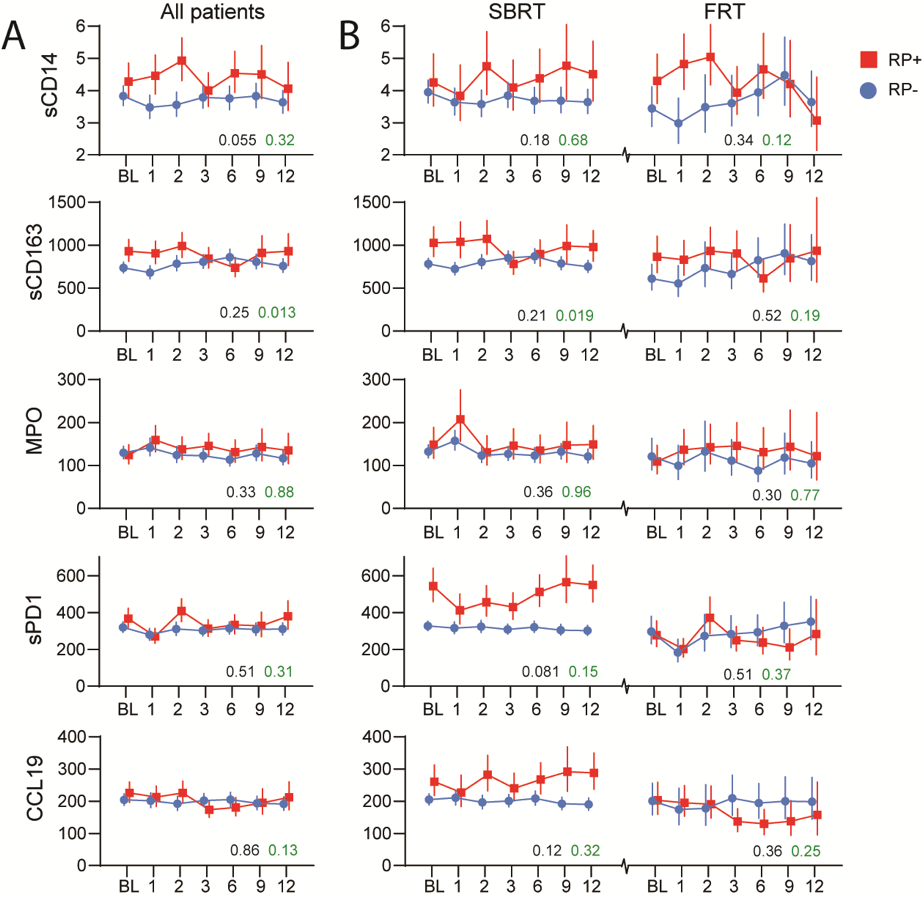

Supplement: Supplementary file 1 [file DataSheet_1.docx]
